# Identifying cell-type-specific spatially variable genes with ctSVG

**DOI:** 10.21203/rs.3.rs-5655066/v1

**Published:** 2024-12-19

**Authors:** Haotian Zhuang, Xinyi Shang, Wenpin Hou, Zhicheng Ji

**Affiliations:** 1 Department of Biostatistics and Bioinformatics, Duke University School of Medicine, Durham, NC, USA.; 2 Department of Biostatistics, Columbia University Mailman School of Public Health, New York City, NY, USA.

## Abstract

Spatially variable genes (SVGs) reveal the molecular and functional heterogeneity of cells across different spatial regions of a tissue. We found that sample-wide SVGs, identified by previous methods across the whole sample, largely overlap with cell-type marker genes derived from single-cell gene expression, leaving the spatial location information largely underutilized. We developed ctSVG, a computational method specifically tailored for Visium HD spatial transcriptomics at single-cell resolution. ctSVG accurately assigns Visium squares to cells and identifies cell-type-specific SVGs. We show that cell-type-specific SVGs identified by ctSVG include many new genes that do not overlap with sample-wide SVGs or cell-type marker genes, and that these genes reveal important biological functions in real spatial datasets.

## Introductions

Spatial transcriptomics (ST) technologies, which measure both gene expression and the spatial locations of cells, enable the study of how gene expression patterns change spatially across tissue regions. Identifying spatially variable genes (SVGs) is crucial for understanding the spatial heterogeneity of tissue structures and organization. Several computational methods have been developed to identify sample-wide SVGs across all cells in an ST sample, including SpatialDE^1^, SPARK^2^, Giotto^3^, nnSVG^4^, MERINGUE^5^, and PreTSA^6^. These methods have been applied to study the spatial heterogeneity of gene expression in various tissues, such as breast cancer and the hippocampus^2^.

**Figure 1.**
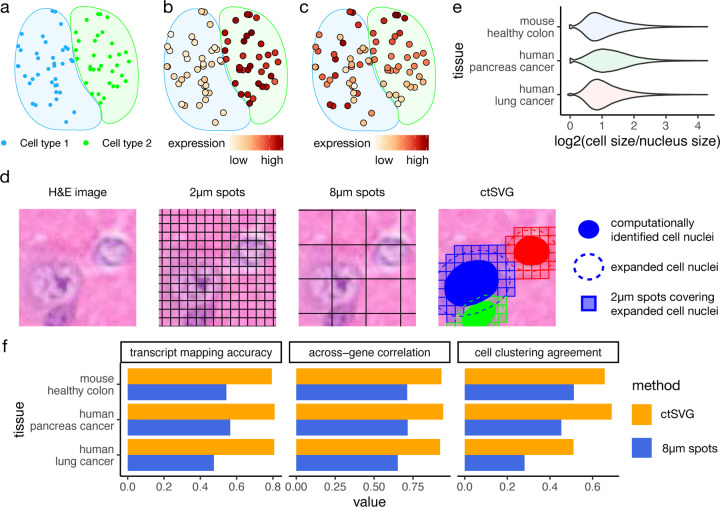
**a-c**, A schematic example showing the spatial distribution of two cell types (**a**), the expression of a sample-wide SVG that is also a marker gene for cell type 2 (**b**), and the expression of a cell-type-specific SVG in cell type 2, with a spatial expression pattern that decreases vertically (**c**). **d**, An example spatial region showing the original H&E image, 2*µ*m spots generated by Visium HD, the 8*µ*m spots approach, and the expanded cell nuclei approach by ctSVG. **e**, Log2 ratios of cell sizes and nuclei sizes for different tissues. **f**, Comparison of transcript mapping accuracy, across-gene correlation, and cell clustering agreement between ctSVG and the 8*µ*m spots approach.

However, sample-wide SVGs identified by these methods are confounded by the non-uniform spatial distribution of different cell types. In many tissues, such as the brain, cells belong to multiple cell types, and certain cell types are restricted to specific spatial regions. Thus, genes specifically expressed in a given cell type will also exhibit expression patterns specific to the spatial region occupied by that cell type (Figure 1a–b). Since gene expression variation is largely driven by these cell type marker genes, existing methods tend to prioritize these genes as top SVGs. However, such genes can also be identified through differential analysis across cell types in conventional single-cell RNA-seq (scRNA-seq) data and do not necessarily depend on spatial information. Therefore, sample-wide SVGs introduce little new knowledge beyond what has already been learned from scRNA-seq data.

To address this issue, ideally, one needs to identify genes with spatially varying expression patterns within each cell type. These genes may not be cell type marker genes and may not appear at the top of the differential gene list in scRNA-seq analysis (Figure 1c). Identifying such cell-type-specific SVGs will fully unlock the power of ST, leading to new insights into the spatially heterogeneous functions of each cell type. However, studying cell-type-specific SVGs has been a challenging task due to the low resolution of ST technologies, such as 10x Visium. While methods such as CTSV^7^, C-SIDE^8^, and spVC^9^ can perform statistical testing and identify spatially varying covariate effects, they cannot be used to directly study and visualize spatial gene expression patterns. In addition, a systematic comparison of sample-wide SVGs, cell-type-specific SVGs, and cell type marker genes is lacking.

The newly developed 10x Visium HD platform^10^ represents a major breakthrough in ST technology, providing expression profiles for the whole transcriptome at single-cell resolution. Compared to other ST technologies that do not achieve single-cell resolution (e.g., 10x Visium) or can only reliably measure the expression profiles of a limited number of genes (e.g., 10x Xenium), Visium HD is ideal for identifying cell-type-specific SVGs. However, several obstacles remain in analyzing data from Visium HD. First, Visium HD measures gene expression profiles in 2-micron squares, and this information must be converted into single-cell gene expression before identifying individual cell types and cell-type-specific SVGs. Second, since cell types are inferred computationally from the data, they cannot be treated as fixed, unlike the spatial locations of cells. It is necessary to account for the additional variation introduced by the uncertainty of inferred cell types when statistically modeling cell-type-specific SVGs in a rigorous manner, similar to previous work in pseudotime analysis^11^.

In this study, we developed ctSVG, a computational method to extract single-cell gene expression profiles from Visium HD data and identify cell-type-specific SVGs. We systematically compared sample-wide SVGs identified by previous methods, cell-type-specific SVGs identified by ctSVG, and cell type marker genes across seven Visium HD datasets from different species and tissue types. We also explored the spatial gene expression patterns of identified cell-type-specific SVGs in two tissues. Our results demonstrate the unique advantage of cell-type-specific SVGs in understanding the spatial heterogeneity of cells.

## Results

### ctSVG accurately assigns Visium HD squares to cells

The default analysis pipeline of Visium HD pools 2*µ*m squares into 8*µ*m squares and treats these 8*µ*m squares as the smallest units in downstream analysis. However, these 8*µ*m squares cannot accurately reflect the gene expression profiles of single cells. Figure 1d shows an example where a single cell is captured by multiple 8*µ*m squares, and one 8*µ*m square overlaps with multiple cells. In comparison, ctSVG first performs cell segmentation on the accompanying H&E images to identify the boundaries of cell nuclei, then expands the nuclei boundaries to approximate the whole cell boundaries, and finally pools 2*µ*m squares covering the expanded cell nuclei (Figure 1d, Methods). The computational expansion of cell nuclei is necessary because the whole cell boundary is difficult to directly obtain from H&E images.

We tested the optimal strategy for cell nuclei expansion using 10x Xenium data with multimodal cell segmentation. This dataset contains both whole cell boundaries and cell nuclei boundaries. We found that the area occupied by a whole cell is typically twice the area occupied by its nucleus (Figure 1e). Therefore, in ctSVG, the area of the expanded cell nuclei is set to be twice the area of the original cell nuclei.

We evaluated the performance of Visium HD’s default strategy of 8*µ*m squares and ctSVG using the 10x Xenium data (Figure 1f, Methods). Results obtained through 10x Xenium’s whole cell segmentation are treated as the gold standard. We found that ctSVG is able to correctly assign most transcripts to their corresponding cells with an accuracy of around 80%. The gene expression profiles obtained by ctSVG are highly consistent with the gold standard, as demonstrated by high across-gene correlations. In terms of downstream analysis, cell clustering results obtained by ctSVG also show high agreement with the gold standard. For all three metrics, ctSVG substantially outperforms the 8*µ*m squares, demonstrating that ctSVG more accurately captures single-cell gene expression profiles. These single-cell gene expression profiles can then be processed using pipelines designed for single-cell analysis, such as Seurat^12^ and GPTCelltype^13^, for identifying cell clusters and cell types.

### Cell-type-specific SVGs identified by ctSVG provide new biological insights

**Figure 2.**
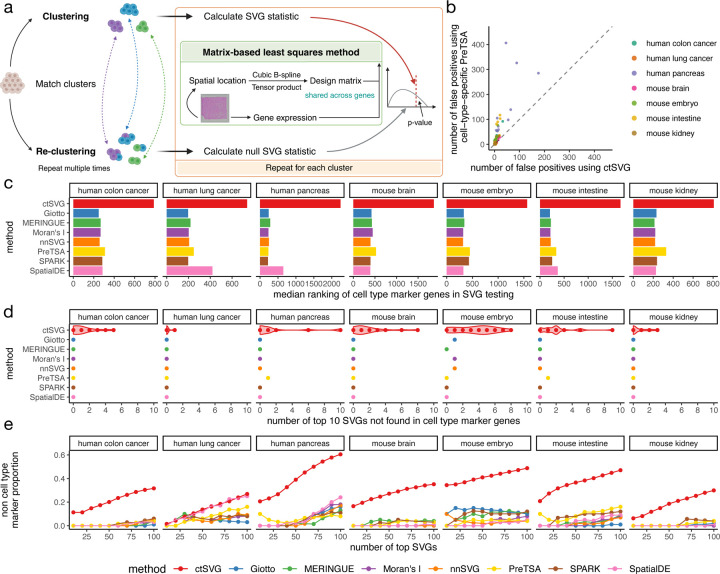
**a,** Framework of ctSVG for identifying cell-type-specific SVGs. **b,** Number of false positives identified by ctSVG, or by PreTSA in each cell type separately, in a null simulation study. **c,** Median ranking of all cell type marker genes in SVG results obtained by each method. For ctSVG, the median of SVG results across all cell types is shown. **d,** Number of top 10 SVGs identified by each method that are not cell type marker genes. For ctSVG, each dot represents the result in one cell type. **e,** The proportion of top SVGs identified by each method that are not cell type marker genes. For ctSVG, the average proportion across cell types is shown.

Next, ctSVG uses a computationally efficient approach to identify cell-type-specific SVGs (Figure 2a), based on our previous work with PreTSA^6^. We have demonstrated that PreTSA is the only method capable of fitting and testing SVGs for large ST datasets in a reasonable amount of time^6^. For each cell cluster, ctSVG fits a B-spline regression model to capture the spatial expression pattern of a gene. Since the spatial location of a cell is fixed, these regression models share the same design matrix, enabling ctSVG to perform all computations related to the design matrix once, greatly increasing computational efficiency. Unlike PreTSA, ctSVG reassigns cells to clusters in the statistical testing step. This non-parametric approach allows ctSVG to account for additional statistical variance induced by computationally inferred cell clusters, similar to pseudotime analysis^11^. In a null simulation study, where the cell locations were randomly permuted, the non-parametric strategy used by ctSVG substantially reduces false positives compared to the parametric strategy used by PreTSA, which does not account for the additional variance (Figure 2b).

We then evaluated whether cell-type-specific SVGs identified by ctSVG can reveal new genes that are not cell type marker genes in three human tissues and four mouse tissues. Seven sample-wide SVG methods were also included for comparison. Cell type marker genes are defined as differential genes compared across cell clusters (Methods). Compared to ctSVG, sample-wide SVG methods have a substantially higher tendency to rank cell type markers as top SVGs (Figure 2c). In almost all cases, the top 10 sample-wide SVGs are all cell type marker genes, whereas the top 10 SVGs identified by ctSVG include many new genes that are not cell type marker genes (Figure 2d). A similar trend holds for larger numbers of top SVGs (Figure 2e). While around 40% of the top 100 SVGs identified by ctSVG are new genes in many tissues, only around 10% of the top 100 sample-wide SVGs are new genes in most tissues. These results suggest that sample-wide SVGs are highly consistent with cell type marker genes, whereas ctSVG can identify new genes that cannot be discovered by simply comparing gene expression across cell clusters.

### Cell-type-specific SVGs in mouse embryo

**Figure 3.**
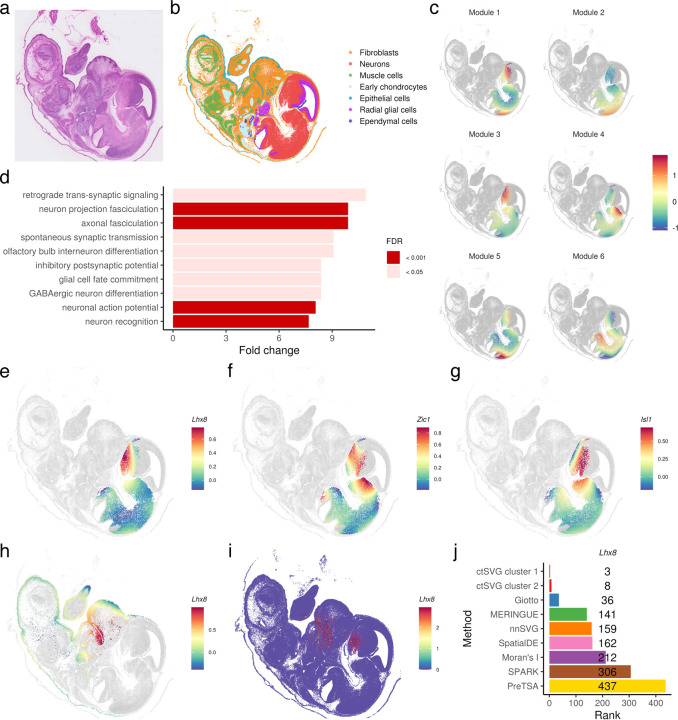
**a,** H&E image of the mouse embryo tissue. **b,** Cell type annotations based on unsupervised clustering and marker genes. **c,** Spatial gene expression pattern of metagenes for each gene module in cell cluster 1 (neurons). **d,** Top GO terms enriched in gene module 3 of cell cluster 1. **e-g**, Fitted spatial gene expression patterns of *Lhx8* (**e**), *Zic1* (**f**), and *Isl1* (**g**) in cell cluster 1. **h**, Fitted spatial gene expression pattern of *Lhx8* in cell cluster 2 (fibroblasts). **i**, Spatial gene expression pattern of *Lhx8* across all cells. **j**, Ranking of *Lhx8* across different SVG methods.

After identifying cell-type-specific SVGs, ctSVG provides comprehensive functions for visualizing and analyzing these genes as part of downstream analysis. As an example, we applied ctSVG to a Visium HD dataset from an E15.5 mouse embryo (Figure 3a). Using ctSVG, we extracted single-cell gene expression profiles, performed cell clustering, and identified seven cell types based on marker genes (Figure 3b, Supplementary Figures 1–2). For each cell cluster, ctSVG organizes cell-type-specific SVGs into distinct gene modules and identifies enriched gene ontology (GO) terms for each module. Additionally, ctSVG visualizes the spatial gene expression patterns of each module through a metagene, constructed by averaging the fitted gene expression values within each module (Figure 3c).

The gene modules identified by ctSVG align well with known biology in the mouse embryo. For example, consider cell cluster 1, which represents neurons (Figure 3c–g). The metagene of gene module 3 shows substantially higher expression levels in spatial regions corresponding to the head (Figure 3c). Consistent with neuronal development, GO terms enriched in gene module 3 include neuron projection fasciculation, axonal fasciculation, and olfactory bulb interneuron differentiation (Figure 3d). Many of the top-ranked cell-type-specific SVGs in gene module 3 are also reported to be associated with neuronal development, including *Lhx8*^14,15^, *Zic1*^16–18^, and *Isl1*^19–21^ (Figure 3e–g).

We further investigated the function of *Lhx8*, a gene critical for the formation of forebrain cholinergic neurons^14^ and associated with tooth development^22^. ctSVG ranked *Lhx8* as the third and eighth most differential gene in cell cluster 1 (neurons) and cell cluster 2 (fibroblasts), respectively. In neurons, *Lhx8* shows high expression in the head region (Figure 3e), while in fibroblasts, it exhibits high expression in the mouth region (Figure 3h). These expression patterns are consistent with the known biological functions of *Lhx8*. In comparison, these cell-type-specific patterns are masked when examining spatial gene expression across the entire sample (Figure 3i), and *Lhx8* does not appear among the top 100 SVGs identified by most sample-wide SVG methods (Figure 3j). These results further demonstrate that cell-type-specific SVGs identified by ctSVG can reveal new biological insights that sample-wide SVG methods may overlook.

### Cell-type-specific SVGs in human colon cancer

**Figure 4.**
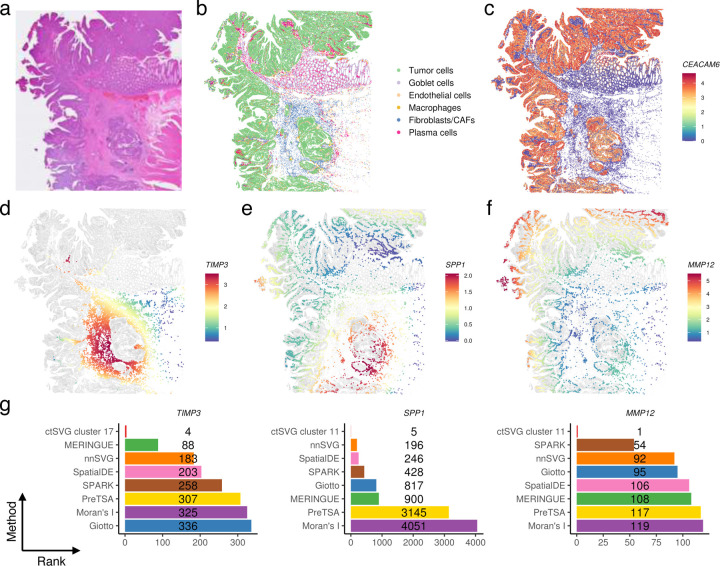
**a,** H&E image of the human colon cancer tissue. **b,** Cell type annotations based on unsupervised clustering and marker genes. **c,** Spatial gene expression pattern of the tumor marker *CEACAM6*. **d,** Fitted spatial gene expression pattern of *TIMP3* in cell cluster 17 (fibroblasts/CAFs). **e-f**, Fitted spatial gene expression patterns of *SPP1* (**e**) and *MMP12* (**f**) in cell cluster 11 (macrophages). **g**, Rankings of *TIMP3*, *SPP1*, and *MMP12* across different SVG methods.

As another example, we used ctSVG to identify cell-type-specific SVGs related to tumor progression in a human colorectal cancer (CRC) sample (Figure 4a). After processing the data with ctSVG, we identified six cell types, including tumor cells, based on marker genes (Figure 4b, Supplementary Figures 3–4). The spatial locations of tumor cells align with the H&E image (Figure 4a) and the expression of *CEACAM6*, a tumor marker gene (Figure 4c). The tumor cells were spatially concentrated in two main areas, referred to as the central and peripheral tumor regions.

Among the top-ranked cell-type-specific SVGs identified by ctSVG, many have been reported to play roles in tumor. For instance, ctSVG ranked *TIMP3* as the fourth most significant gene in cell cluster 17, a fibroblast/CAF cell cluster (Supplementary Figure 3). *TIMP3* shows higher expression in cells situated between the central and peripheral tumor regions, suggesting its potential role in tumor suppression (Figure 4d). This finding aligns with previous research showing that *TIMP3* inhibits matrix metalloproteinases, preventing extracellular matrix degradation and subsequent tumor invasion. This activity is crucial for maintaining structural integrity between tumor clusters to avoid their spread and merging^23,24^.

We further examined the spatial heterogeneity of immune responses in the central and peripheral tumor regions, focusing on macrophages, the most abundant immune cell type in this dataset (Figure 4b). ctSVG identified *MMP12* and *SPP1* as two top-ranked cell-type-specific SVGs in a macrophage cluster (cell cluster 11, Supplementary Figure 3). When expressed in macrophages, *MMP12* may inhibit intestinal tumor growth by influencing macrophage polarization^25^. Conversely, *SPP1*+ macrophages in CRC can promote immune evasion and tumor progression by supporting a desmoplastic tumor structure through interactions with *FAP*+ fibroblasts^26^. We observed that macrophages nearer the central tumor region expressed higher levels of *SPP1* and lower levels of *MMP12*, while macrophages closer to the peripheral tumor region exhibited the opposite expression pattern of *SPP1* and *MMP12* (Figures 4e–f). These findings suggest that the central tumor region is more likely to be an immunosuppressive environment promoting tumor growth, whereas tumor growth may be inhibited in the peripheral tumor region.

Similar to the previous example, *TIMP3*, *SPP1*, and *MMP12* do not appear among the top 100 SVGs in most sample-wide SVG methods (Figure 4g).

## Conclusions

In this study, we developed ctSVG, a computational tool for processing Visium HD data and identifying cell-type-specific SVGs. We demonstrated that while sample-wide SVGs largely overlap with cell type marker genes, cell-type-specific SVGs introduce many new genes that are not found among cell type marker genes. We further showed that these cell-type-specific SVGs are important for understanding the molecular and functional heterogeneity of cell types across spatial regions. Beyond Visium HD data, ctSVG can also be applied broadly to other types of ST data with single-cell resolution, such as 10x Xenium.

## Methods

### ctSVG

#### Input data

For analyzing 10x Visium HD data, ctSVG requires two inputs. The first input is the output from the standard 10x Space Ranger pipeline. The second input is the nuclei segmentation results obtained by running segmentation methods, such as StarDist^27^, on the H&E images accompanying the Visium HD data.

For platforms other than 10x Visium HD, ctSVG can identify cell-type-specific SVGs without performing the data processing steps specific to Visium HD. In this case, ctSVG requires only the gene expression count matrix and a matrix of cell spatial coordinates as inputs.

#### Obtaining aggregated single-cell gene expression profiles

ctSVG first filters out cell nuclei with abnormally large sizes. The area of each segmented cell nucleus is calculated using the sf package (version 1.0–16) in R, and then log-transformed. A cutoff is determined as the mean of the log-transformed areas across all nuclei, plus two times the standard deviation of the log-transformed areas. Nuclei with log-transformed areas larger than this cutoff are filtered out.

ctSVG then assigns each Visium 2*µ*m square to the cell nucleus it overlaps with. For a 2*µ*m square that overlaps with multiple nuclei, the square is uniquely assigned to the nucleus with the largest area of overlap. Note that, for computational efficiency, the area of overlap is approximated. Specifically, each square is divided into 100 subsquares, and the area of overlap is estimated by the number of subsquares that the nucleus overlaps with.

Next, ctSVG expands each cell nucleus to approximate its whole cell boundary, so that the area of the expanded nucleus is twice that of the original nucleus. The ratio of areas between the expanded and original nucleus can be optionally specified by the user. To perform the expansion, the centroid of each nucleus is first calculated using the st_centroid function from the sf package. Denote the coordinates of the centroid as $\left(x_{0},y_{0}\right)$, and the coordinates of the N contour points that define the segmentation as $\left(x_{i},y_{i}\right)$, where i=1,...,N. The coordinates of the expanded contour points are calculated as $\sqrt{r}\left(x_{i}-x_{0}\right)+x_{0}$ and $\sqrt{r}\left(y_{i}-y_{0}\right)+y_{0}$, where r is the ratio of areas between the expanded and original nucleus.

For any 2*µ*m square that has not yet been assigned to a cell, ctSVG repeats the square assignment procedure described above, this time using the expanded cell nuclei.

After the squares have been assigned to cells, ctSVG identifies and removes three types of abnormal cells. A cell is considered abnormal if no 2*µ*m square is assigned to its nucleus, if the total area covered by all assigned 2*µ*m squares is less than half of the cell’s area, or if the assigned 2*µ*m squares are disconnected. Note that these abnormal cells are rare in real datasets.

Finally, ctSVG generates the single-cell gene expression count matrix. For each gene, ctSVG aggregates the gene expression counts across all 2*µ*m squares assigned to a cell to obtain that cell’s gene expression profile.

#### Processing gene expression data

Seurat (version 4.4.0) was used to process the single-cell gene expression count matrix. Specifically, cells with positive expression in at least 300 genes were retained, and genes with positive expression in at least 1% of all retained cells were kept. The log-normalized gene expression matrix was obtained using the NormalizeData function with default settings. Highly variable genes were identified using the FindVariableFeatures function with default parameters. The matrix was scaled using the ScaleData function. PCA was performed using the RunPCA function. Cell clustering was conducted using the FindNeighbors function on the top 10 PCs, followed by FindClusters with the resolution set to 1.2.

#### Removing spatially isolated cells

Before fitting and testing cell-type-specific SVGs, ctSVG filters out cells that are spatially distant from all other cells within each cell cluster. This filtering is done separately for each cell cluster. First, the Euclidean distance from each cell to all other cells within the same cell cluster is calculated. The isolation score of a cell is then defined as the average Euclidean distance to the 1% nearest cells (with a maximum of 50 cells and a minimum of 10 cells). Cells with an isolation score greater than the mean isolation score across all cells, plus six times the standard deviation, are removed.

Finally, genes with positive expression in at least 1% of all retained cells within each cell cluster are kept. Note that this gene filtering was not applied in the analyses shown in Figure 2 to ensure the results of ctSVG are consistent with those of the sample-wide SVGs.

#### Identifying cell-type-specific SVGs

ctSVG uses the same approach as PreTSA to fit the spatial expression pattern of a gene. The details of estimating the fitted gene expression values and associated test statistics were described in our previous work, PreTSA^6^. For the readers’ convenience, we briefly introduce the fitting procedure here.

ctSVG sequentially fits the following regression model for each cell cluster. Let Y be the m×n gene expression matrix, where m represents the number of genes and n represents the number of cells in a given cell cluster, with $y_{\mathrm{ij}}$ denoting the expression level of gene i in cell j. Let $S_{j}=\left(s_{j1},s_{j2}\right)$ represent the 2-dimensional spatial coordinates of cell j.

For each gene i, ctSVG models its expression values across spatial locations as a functional surface:

$$y_{ij}=\beta_{i0}+\sum_{k_{1}=1}^{K+3}b_{1,k_{1}}\left(s_{j1}\right)\beta_{i,k1,0}+\sum_{k_{2}=1}^{K+3}b_{2,k_{2}}\left(s_{j2}\right)\beta_{i,0,k_{2}}+\sum_{k_{1}=1}^{K+3}\sum_{k_{2}=1}^{K+3}b_{1,k_{1}}\left(s_{j1}\right)b_{2,k_{2}}\left(s_{j2}\right)\beta_{i,k_{1},k_{2}}+\varepsilon_{ij}\;\varepsilon_{ij}\overset{\text{iid}}{\sim }N\left(0,\sigma_{i}^{2}\right).$$

Here, $b_{d,1}(s),\ldots ,b_{d,K+3}(s)$ represent the K+3 cubic B-spline basis functions for each dimension $\left(d=1,2\right)$, where K is the number of equidistant internal knots used to define the cubic B-spline bases. The parameters $\beta_{i0},\;\beta_{i,1,0},\ldots ,\beta_{i,K+3,0},\;\beta_{i,0,1},\ldots ,\beta_{i,0,K+3},\;\beta_{i,1,1},\ldots ,\beta_{i,K+3,K+3},$ and $\sigma_{i}^{2}$ are all unknown and will be estimated using the least squares method. An F statistic is subsequently calculated for the fitted model as the test statistic.

To test the statistical significance of cell-type-specific SVGs, ctSVG applies a permutation test to account for the additional variation in computationally inferred cell clusters. Specifically, cell clustering is redone by changing the random.seed parameter to 1, 2, … , 1, 000 in the FindClusters function. A Jaccard index is calculated between each original cluster and each reassigned cluster. For each original cluster, the reassigned cluster with the highest Jaccard index is retained. This process is repeated 1,000 times, and for each original cluster, the 100 reassigned clusters with the highest Jaccard indices are selected.

For each original cluster, the spatial locations of cells in its corresponding reassigned cluster are randomly permuted. The same fitting approach is then applied to the reassigned and permuted cluster to obtain the null test statistic for each gene. This step is repeated for each of the 100 reassigned clusters to generate 100 null test statistics. To enhance numerical accuracy, a Gamma distribution is fitted to these 100 null test statistics using the R package fitdistrplus (version 1.1–11). The *p*-value is calculated as the tail probability of the fitted Gamma distribution exceeding the test statistics calculated from the original data. All *p*-values are then adjusted for multiple testing using the Benjamini-Hochberg (BH) procedure to obtain false discovery rates (FDRs)^28^. By default, an FDR of ≤ 0.05 is used as the significance cutoff.

#### Gene clustering and functional analysis

In each cell cluster, the fitted values of each cell-type-specific SVG across all cells are standardized to have a mean of zero and a standard deviation of one. These cell-type-specific SVGs are then grouped into different gene modules based on their spatial patterns using k-means clustering. The number of modules is automatically determined based on the proportion of the within-cluster sum of squares to the total sum of squares, using findPC^29^ with default settings.

In each gene module, GO enrichment is performed using the R package topGO (version 2.56.0). All *p*-values are then adjusted for multiple testing using the BH procedure to obtain FDRs^28^. GO terms with an FDR of ≤ 0.05 are retained and then ordered in decreasing order by fold change.

### Evaluation of aggregated single-cell gene expression

#### Xenium datasets

Three Xenium datasets were downloaded directly from the 10x Genomics website (https://www.10xgenomics.com/datasets): human lung cancer, human pancreas cancer, and mouse colon. These Xenium datasets include both whole cell segmentation and cell nuclei segmentation results.

Since the original Xenium datasets are quite large, we selected a rectangular region in the middle of each image for computational efficiency. The center of the rectangle corresponds to the center of the original image, and the width and height of the rectangle are 20% of the original image’s width and height, respectively. Only cells within this rectangle are considered in the subsequent analysis.

Transcripts labeled as “NegControlProbe”, “NegControlCodeword”, and “UnassignedCodeword” were all removed.

#### Performing ctSVG and competing method

Instead of performing nuclei segmentation on H&E images, ctSVG directly uses the nuclei segmentation results provided by the original Xenium datasets. To mimic the dataset generated by Visium HD, the entire image was split into consecutive, non-overlapping 2*µ*m squares. The remaining steps of the ctSVG pipeline were then performed to assign the 2*µ*m squares to cells.

For Visium HD’s default strategy of using 8*µ*m squares, the entire image was split into consecutive, non-overlapping 8*µ*m squares. Each cell was assigned to the 8*µ*m square with the largest area of overlap with the cell. An approximation method similar to that used in ctSVG was applied to calculate the area of overlap.

#### Evaluation of transcript mapping accuracy

In the original Xenium datasets, each transcript is already assigned to a cell if it falls within the cell’s whole cell boundary. A transcript is unassigned if it is not within any cell boundary. This information is treated as the gold standard.

Transcript mapping accuracy was calculated as the proportion of transcripts with matching assignments (either both assigned to the same cell or both unassigned) between the gold standard and the method being evaluated.

Note that with the 8*µ*m square method, a square can be assigned to multiple cells. Consequently, a transcript within such a square may also be assigned to multiple cells, complicating the evaluation of assignment agreement. To address this, we arbitrarily assigned the square to a randomly selected cell from the original list of cells it was assigned to, ensuring a one-to-one mapping between squares and cells. Transcript mapping accuracy was calculated afterward. This operation was not performed in other evaluations discussed below.

#### Evaluation of across-gene correlation and cell clustering agreement

For both ctSVG and the 8*µ*m square method, single-cell gene expression matrices were obtained by counting the number of RNA transcripts falling within the squares assigned to each cell. The gene expression count matrices from the original Xenium data, ctSVG, and the 8*µ*m square method were then processed using Seurat, following the same procedure as in the standard ctSVG pipeline described above. The only differences were that cells with at least 10 total reads were retained, and the scale.factor parameter in the NormalizeData function was set to 100.

The across-gene correlation was calculated using the scaled and log-normalized gene expression values. For each cell, a Pearson correlation coefficient was computed across genes between the original Xenium gene expression and the gene expression from either ctSVG or the 8*µ*m square method. The median correlation coefficient across all cells was then taken.

Cell clustering agreement was calculated using the adjusted Rand index (via the adjustedRandIndex function in the mclust R package) between the cell clustering obtained from the original Xenium gene expression and the cell clustering obtained from either ctSVG or the 8*µ*m square method.

### Evaluation of cell-type-specific SVGs

#### Visium HD datasets

Seven Visium HD datasets were downloaded directly from the 10x Genomics website (https://www.10xgenomics.com/datasets): human colorectal cancer, mouse small intestine, human lung cancer, mouse brain, human pancreas, mouse embryo, and mouse kidney.

All Visium HD datasets were processed using the standard ctSVG pipeline described above.

#### Cell type marker gene identification

Genes with differential expression between cells from one cluster and all other cells were identified using Seurat’s FindAllMarkers function, with the max.cells.per.ident parameter set to 5000. In each cluster, cell type marker genes were identified as the 50 genes with the smallest *p*-values. A union set of cell type marker genes was taken across all clusters.

#### Competing methods

Giotto (version 1.0.4), MERINGUE (version 1.0), the RunMoransI function in Seurat (version 4.4.0), nnSVG (version 1.7.4), PreTSA (version 1.1), the Gaussian version of SPARK (version 1.1.1), and SpatialDE (version 1.1.3) were used to identify SVGs with default settings.

PreTSA and ctSVG were applied directly to the original datasets. All other methods were performed on subsets of the original datasets, where 10,000 cells were randomly selected for each dataset. This was necessary because the original datasets were too large for methods other than PreTSA and ctSVG to complete in a reasonable time.

## Supplementary Material

Supplement 1

## Data Availability

All datasets used in this study were downloaded from the 10x website (https://www.10xgenomics.com/datasets). The R package ctSVG, along with a detailed user manual, is publicly available at https://github.com/haotian-zhuang/ctSVG. Figure 2a was created using BioRender (BioRender.com).
