# Supplementary material for: Identifying cell-type-specific spatially variable genes with ctSVG
Source: Res Sq. 2024 Dec 19:rs.3.rs-5655066. Preprint. [Version 1] doi: 10.21203/rs.3.rs-5655066/v1 (PMC11702777; doi:10.21203/rs.3.rs-5655066/v1)
Supplement: Supplement 1 [file NIHPPRS5655066v1-supplement-1.pdf]

Supplementary Files

This is a list of supplementary files associated with this preprint. Click to download.

- supp.pdf
